# Optimizing the Y Content of Welding Wire for TIG Welding of Sand-Cast Mg-Y-RE-Zr Alloy

**DOI:** 10.3390/ma18194549

**Published:** 2025-09-30

**Authors:** Yikai Gong, Guangling Wei, Xin Tong, Guonan Liu, Yingxin Wang, Wenjiang Ding

**Affiliations:** National Engineering Research Center of Light Alloy Net Forming and State Key Laboratory of Metal Matrix Composites, School of Materials Science and Engineering, Shanghai Jiao Tong University, Shanghai 200240, China; yikai-g@sjtu.edu.cn (Y.G.); weigl010@sjtu.edu.cn (G.W.); xintong@sjtu.edu.cn (X.T.); guonan_l@sjtu.edu.cn (G.L.); wjding@sjtu.edu.cn (W.D.)

**Keywords:** Mg-Y-RE-Zr alloy, TIG welding, microstructure, mechanical properties, grain size

## Abstract

The widespread application of WE43 (Mg-4Y-2Nd-1Gd-0.5Zr) alloy castings in aerospace components is hindered by the frequent formation of defects such as cracks, pores, and especially yttria inclusions. These defects necessitate subsequent welding. However, using homologous WE43 filler wires often exacerbates these issues, leading to high crack susceptibility and reintroduction of inclusions. Herein, we propose a novel strategy of tailoring Y content in filler wires to achieve high-quality welded joint of WE43 sand castings. Systematic investigations reveal that reducing Y content to 2 wt.% (WE23) effectively suppresses oxide inclusion formation and significantly enhances the integrity of the joint. The fusion zone microstructure evolves distinctly with varying Y levels: grain size initially increases, peaking at 24 μm with WE43 wire, then decreases with further Y addition. Moreover, eutectic compounds transition from a semi-continuous to a continuous network structure with increasing Y content, deteriorating mechanical performance. Notably, joints welded with WE23 filler exhibit minimal performance loss, with ultimate tensile strength, yield strength, and elongation reaching 93.0%, 98.0%, and 97.4% of the sand-cast base metal, respectively. The underlying strengthening mechanisms and solute-second phase relationships are elucidated, highlighting the efficacy of optimizing Y content in welding wire design. This study provides valuable insights toward defect-free welding of high-performance Mg-RE alloy castings.

## 1. Introduction

Magnesium (Mg) alloys are gaining increasing recognition across a wide range of industries due to their lightweight nature. Among these, the Mg-Y-RE-Zr (WE) series of alloys exhibits excellent ability to withstand high temperatures, vibrations, and corrosive environments. In the industry, sand casting is used to manufacture aerospace components due to its ability to produce large and complex-shaped castings, making it a suitable candidate for various applications. Therefore, WE43 alloys are extensively employed in large structural components such as aero-engine gearboxes and helicopter transmission systems [1,2,3,4].

However, the extended solidification time in sand casting further increases the likelihood of defects, such as porosity and cracks in Mg alloy castings, ultimately impairing their performance [5,6]. Furthermore, as the Y element exhibits a stronger tendency to oxidize compared to Mg, it is more likely to form oxidation slags within the Mg-Y-RE-Zr alloy [7,8]. Therefore, an effective welding technique should be developed to enhance the performance of welded joints. TIG welding is one of the most prevalent welding techniques. Although TIG welding has disadvantages including larger heat input and lower welding heat density, it can be operated manually and offers greater adaptability [9,10]. It offers unique advantages for welding complex geometries such as arcs and internal cavities in magnesium alloy components compared to some advanced welding methods such as friction stir welding (FSW) [11], diffusion welding (DW) [12], and laser beam welding (LBW) [13].

To date, previous studies on the TIG welding of Mg-Y-RE-Zr alloys primarily focused on welding wires made from the same material [14,15,16]. However, hot cracking is likely to occur when using the same type of welding wire. This is especially so for WE43 alloys with high Y content, using the same type of welding wire can readily lead to the formation of new Y_2_O_3_ inclusions in the molten pool, leading to the performance degradation of the welded joint [17,18,19].

Previous research has shown that increasing Y content in Mg-RE alloys leads to a reduction in grain size and microsegregation at the grain boundary, which improves the overall strength [20,21,22,23]. However, excessive Y content leads to the formation of an interconnected second phase, which is detrimental to alloy properties. The oxide layer for Mg-RE cannot be easily broken, which can impair the performance of the as-cast alloy [24]. It is crucial to achieve a balance between properties and manufacturability. Although abundant research on the effects of Y has been conducted on Mg-RE alloys, studies focusing on the role of Y in welding wires are lacking.

Based on the literature, TIG welding was performed in this study using filler wires with varying Y content, and the properties of the resultant joints were evaluated in terms of both microstructure and mechanical characteristics. The results demonstrate theoretical progress in the welding metallurgy of Mg-Y-RE-Zr alloys. This understanding is crucial for aerospace applications, where such welded components must meet stringent performance requirements under demanding service conditions.

## 2. Materials and Methods

### 2.1. Preparation of the Gravity Sand-Cast Alloy

**Figure 1:**
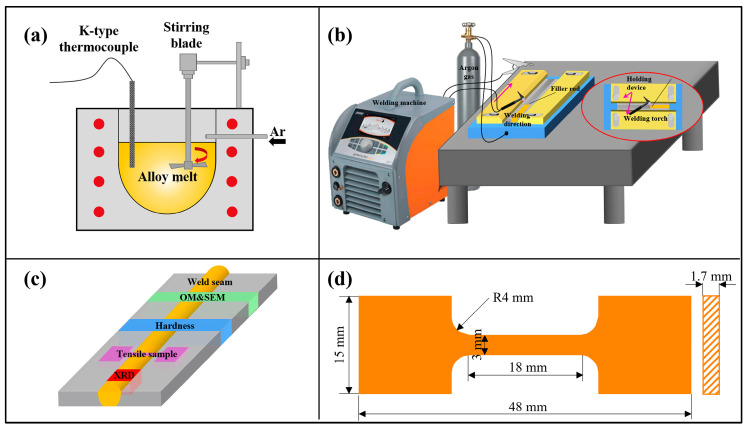
Schematic illustration indicating the experimental setup for (**a**) gravity sand casting process, (**b**) welding process, (**c**) sampling location, and (**d**) geometry of the tensile specimen (obtained 2 mm below the surface of the weld plate).

In this study, both the welding wire and the base material were produced by gravity sand casting, as shown in Figure 1a. Mg-xY-2Nd-1Gd-0.5Zr alloys were prepared by melting commercially pure magnesium (>99.95%) together with other master alloys in a crucible under a protective atmosphere of CO_2_ (99 vol%) and SF_6_ (1 vol%). After melting, the alloys were refined to ensure the complete dissolution of rare earth elements and were subsequently poured into preheated sand molds to yield rectangular plates. Filler wires with a diameter of 5 mm were prepared by direct wire cutting from sand-cast Mg-xY-2Nd-1Gd-0.5Zr alloy plates. The wires were designated WE03, WE23, WE43, and WE63 based on their nominal Y contents (0 wt.%, 2 wt.%, 4 wt.%, and 6 wt.%, respectively). The actual chemical composition of the wires was verified using inductively coupled plasma atomic emission spectroscopy (ICP-AES), as listed in Table 1. Detailed information has been provided in our previous studies [18,19].

### 2.2. Welding Process

Rectangular plates (100 mm × 100 mm × 5 mm) were prepared using wire cutting, with a 90° single-V groove configuration and a 1 mm unbeveled root face. The 5 mm thick plates were selected to simulate sand-cast Mg-Y-RE-Zr components of the same thickness in repair welding scenarios. A 90° single-V groove was machined to replicate the geometry of excavated cavities after defect removal in industrial repair practices. All workpiece surfaces and filler wires were ground with 400# SiC sandpaper and degreased in acetone. One-sided welding of the WE43 magnesium alloy was performed using a Rehm INVERTIG.PRO digital 450 AC/DC system (Figure 1b). Before welding, the WE43 magnesium plates were clamped in a steel fixture to minimize distortion caused by thermal expansion. The specific parameters of the welding process are listed in Table 2. Note that the welding parameters used in this study are based on our previous studies [18,19].

### 2.3. Characterization

The sampling position of the welded plate is illustrated in Figure 1c. The joints were analyzed using X-ray real-time image detect system, with the base metal as the control group. For microstructural characterization, welded joint specimens were prepared by mechanical grinding, polishing, and etching in a solution of 4.2 g picric acid, 10mL acetic acid, 10 mL water, and 70 mL ethanol. Microstructural characterization was performed using an optical microscope (Axio Observer A1, Precise instrument CO., Ltd., Beijing, China) and scanning electron microscopy (RISE-MAGNA, TESCAN, Brno-Kohoutovice, Czech Republic) combined with energy dispersive spectrometry (EDS). Notably, EDS point analysis was performed on the magnesium matrix within the FZ for each set of 10 randomly selected points. Electron backscatter diffraction (EBSD) on the SEM was used to analyze the crystalline microstructure and grain size distribution of the welded joints. Low angle grain boundaries (LAGBs) were defined as boundaries with misorientation angles below 15°, while high angle grain boundaries (HAGBs) were defined as those with angles above 15°. The phase composition was characterized by X-ray diffraction (D8 ADVANCE Da Vinci, Brook, Germany). The Vickers hardness profile of the welded zone was determined by applying a load of 49 N for 15 s using an HV-300 hardness tester (XHVT–10Z, Shangcai Testing Machine Co., Ltd., Shanghai, China). Tensile specimens were prepared by cutting specimens horizontally 2 mm below the surface of the weld plate and evaluated using a Zwick/Roell Z100 universal testing machine (ZwickRoell, Ulm, Germany) with an extensometer at room temperature at a constant strain rate of 1 mm/min for each set of five parallel tensile specimens. The geometry of tensile specimens is shown in Figure 1d.

## 3. Results

### 3.1. Macrostructure of the Welded Joints

**Figure 2:**
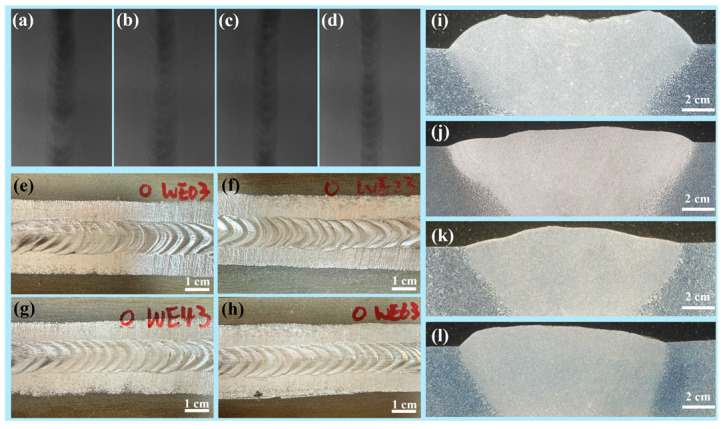
X-ray radiography analysis and macroscopic morphology of welded joints using a welding wire of (**a**,**e**,**i**) WE03, (**b**,**f**,**j**) WE23, (**c**,**g**,**k**) WE43, and (**d**,**h**,**l**) WE63.

The analysis of the welded joints by X-ray real-time image detect system is provided in Figure 2a–d. All welds exhibited good quality with no obvious defects. Since there was no change in heat input (determined by welding current, voltage, and speed), no significant macroscopic differences were observed among the samples. The fusion zone (FZ) appeared darker than the base metal due to its increased thickness, which is consistent with the previous findings for the WE43 alloy [18].

Macrostructures of the welded joints are displayed in Figure 2e–h. The weld morphology exhibited a graceful fish-scale pattern, indicating that the process parameters were optimized. Cross-sectional macrographs in Figure 2i–l show that the overall morphology of the welded joints exhibited minimal variation despite the different Y concentrations in the filler wires. Notably, the thickness of the FZ was greater than that of the base metal and the heat affected zone (HAZ), which coincides with the real-time image.

### 3.2. Microstructure of the Welded Joints

**Figure 3:**
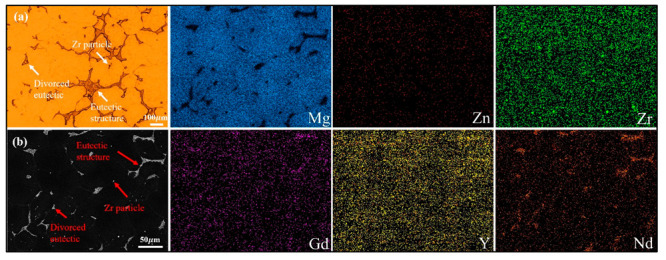
(**a**) OM and (**b**) SEM images and corresponding EDS maps of sand-cast alloy.

The microstructure of the sand-cast WE43 alloy is shown in Figure 3. The as-cast microstructure comprises equiaxed α-Mg dendrites and interdendritic eutectic phases. Divorced eutectics were observed at the triple junction of grain boundaries. SEM combined with EDS confirmed the complex distribution of alloying elements. Zn was uniformly distributed across both the matrix and secondary phases. Zr was evenly distributed in the α-Mg matrix, with some Zr-rich particles dispersed within the α-Mg grains. In contrast, Y, Gd, and Nd were detectable within the α-Mg matrix but were predominantly enriched in the secondary phases.

**Figure 4:**
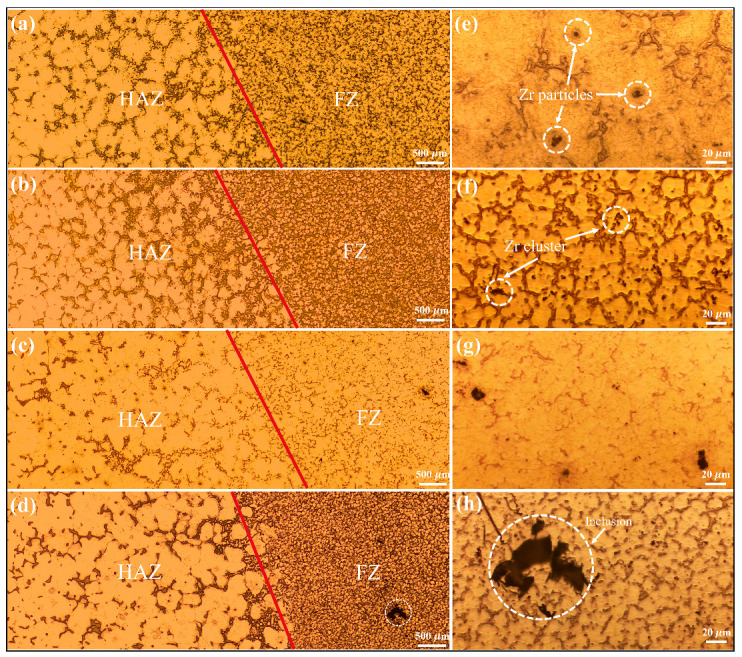
Optical micrographs showing the welding joints and the corresponding magnified views of the FZ for alloys: (**a**,**e**) WE03, (**b**,**f**) WE23, (**c**,**g**) WE43, and (**d**,**h**) WE63.

The optical microstructure of the FZ and HAZ in different joints is illustrated in Figure 4, in which the red lines represent the fusion lines. The grain structure of the FZ was significantly refined compared to the base material (BM), owing to the rapid cooling rate and large temperature gradient [25]. However, the HAZ exhibited coarser grains and a more continuous network of second phases compared to those in FZ. Notably, the proportion of secondary phases in WE43 FZ is much lower. With the increasing Y content, large dark patches could be observed within the FZ; these are believed to be the presence of oxide inclusions [25,26].

**Figure 5:**
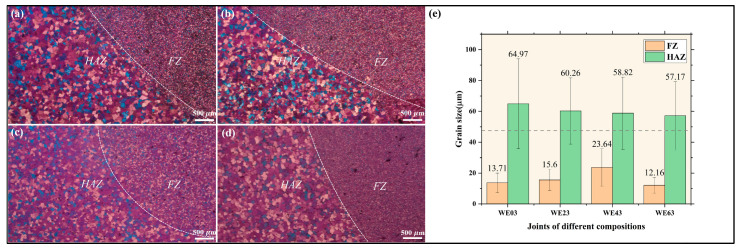
Grain analysis of FZ and HAZ based on OM and EBSD: (**a**–**d**) Optical microstructure of FZ and HAZ formed with WE03, WE23, WE43, and WE63; (**e**) Average grain size analyzed by EBSD.

It is worth mentioning that WE43-welded specimens displayed an anomalous coarsening phenomenon in the FZ, deviating from the typical refinement trend. The polarized light mode in OM was employed in Figure 5a–d to capture different microstructural characteristics, in which the dash lines represent the fusion lines. Figure 5e shows the detailed grain size distribution in FZ using EBSD statistics. The dotted line indicates the average grain size of BM, serving as a reference. The grain size in the FZ varied with increasing Y content: it initially increased, reaching a maximum grain size of 24 µm for the WE43 joint, and then decreased with further Y content. Meanwhile, HAZ showed a continuous grain refinement trend with increasing Y content. Tong et al. performed the repair welding on WE43 Mg alloy using the same filler wire. When the other processing parameter remains constant, the current ranging from 150A to 170A resulted in a grain size range of 20 µm to 13 µm, which is significantly different from our results [18]. This difference is attributed to the variation in heat input and cooling rate between different functional variants of TIG welding. Repair welding aims at a small local molten pool, while butt-welding targets longer welds with greater heat input. In addition, the molten pool in repair welding is surrounded by a three-dimensional base metal, resulting in a faster cooling rate. Although butt welding employs a bevel at the bottom, which assists heat dissipation, it primarily dissipates heat through two-dimensional conduction at both ends [26].

**Figure 6:**
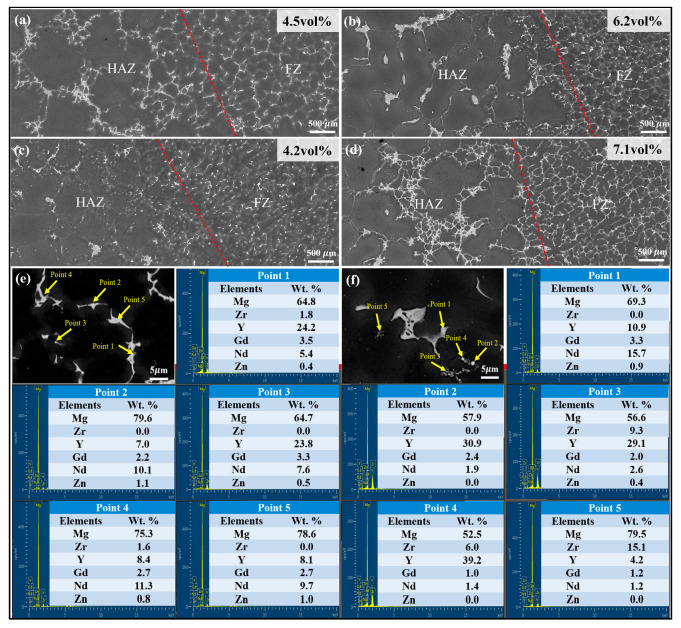
SEM micrographs of the transition zones between HAZ and FZ formed with different content of welding wires: (**a**) WE03, (**b**) WE23, (**c**) WE43, (**d**) WE63, and (**e**,**f**) the EDS results of FZ in (**b**,**d**).

The SEM microstructures of the interface between HAZ and FZ of different joints are displayed in Figure 6a–d, where the red lines represent the fusion lines. Volume fraction of secondary phases in FZ is presented in the top right corner. The comparative microstructural analysis revealed distinct phase distributions across FZ depending on the filler wire content. Welds produced with all filler wires except WE63 exhibited a predominantly semi-continuous distribution of second phases. In contrast, FZ of WE63 showed a continuous and network-like structure of secondary phases. This transition correlated directly with the elevated concentration of rare earth (RE) solutes inherent to the WE63 filler wire. Furthermore, oxide inclusions within the FZ of WE63 remained more pronounced, which is consistent with prior optical microscopy findings.

The representative FZ microstructure, along with the corresponding EDS point scanning analysis, is presented in Figure 6e,f. Divorced eutectic and some cubic phases were observed within the WE23 joint. EDS point analysis in Figure 6e revealed that secondary phases precipitated predominantly along grain boundaries in the WE23 joint were composed chiefly of Nd and Y. Therefore, the major constituent phases within the FZ of WE23 are inferred to be Mg_5_ (Gd, Y) and Mg_12_Nd. Crucially, the cubic phases exhibited significant Y enrichment, as illustrated by representative points 1 and 3. Figure 6f displays the SEM micrograph and EDS analysis of the WE63-welded joint. The EDS point analysis (Points 3 and 5) detected dispersed flocculent clusters within the FZ, composed mainly of Zr and Y. Compared to WE23, the FZ of the WE63-welded joint exhibited much larger lamellar precipitates coexisting with Y-rich cubic phases. These net-like phases were confirmed as Mg_5_ (Gd, Y) in previous study [27]. Moreover, a substantially higher density of Y-rich cubic phases and a larger proportion of Y in second phases are observed in the WE63-welded joint.

**Figure 7:**
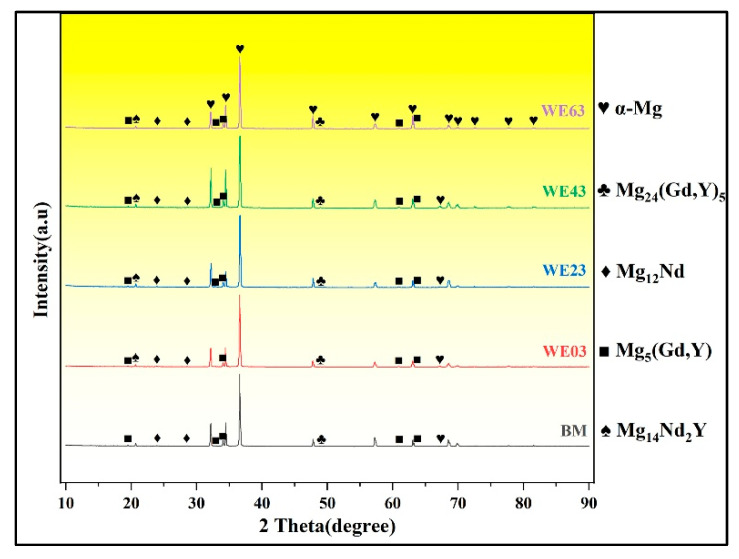
XRD spectra of the BM and FZ in the different joints.

The XRD patterns of BM and the FZ produced with varying Y contents are compared in Figure 7. The results revealed that all samples were characterized by the presence of α-Mg solid solution and intermetallic compounds: Mg_24_ (Gd, Y)_5_, Mg_12_Nd, Mg_14_Nd_2_Y, and Mg_5_ (Gd, Y). The phase composition remained consistent before and after welding, irrespective of Y content. When using WE03 filler wire, the characteristic Mg_5_ (Gd, Y) peak at 2θ = 32.5° disappeared. Corresponding to our findings in SEM, the Mg_5_ (Gd, Y) peak located at 2θ = 63° progressively intensified with increasing Y content, which is consistent with the finding in the literature [28].

**Figure 8:**
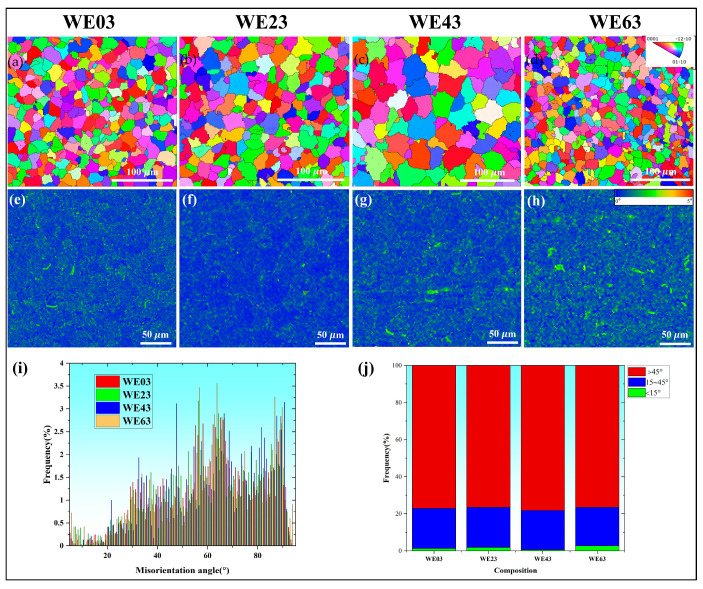
EBSD results of the center of FZ in different joints: (**a**–**d**) The IPF maps; (**e**–**h**) corresponding KAM maps, and (**i**,**j**) misorientation angle distributions.

The inverse pole figure (IPF) maps of the FZ in different joints are displayed in Figure 8a–d. All regions primarily consisted of equiaxed grains, exhibiting no significant grain orientation. The WE43-welded joint displayed abnormal grain coarsening, consistent with the findings in Figure 5. The corresponding kernel average misorientation (KAM) maps are presented in Figure 8e–h. KAM represents the local orientation difference, where regions with characteristic colors correspond to larger local orientation differences. It was observed that the local orientation difference was the smallest when WE43 was used as the welding wire, whereas WE23 and WE63 were characterized by high KAM values. The low KAM value of WE43 joints corresponded to their minimum dislocation density. This was likely due to the matching composition of the weld and base metal, resulting in a uniform overall coefficient of thermal expansion. Consequently, the shrinkage experienced minimal constraint during welding, leading to a more uniform stress distribution. The overall misorientation angle distributions of the different joints are presented in Figure 8i,j. It is generally accepted that LAGBs result from dislocation stacking [2,15]. Consequently, the higher the number of LAGBs, the higher the dislocation density. When the filler wire shifted from WE03 to WE23, the fraction of LAGBs in the FZ rose from 1.3% to 1.8%. However, a further transition from WE23 to WE43 led to a decrease in LAGBs from 1.8% to 0.6%. Subsequently, the change from WE43 to WE63 resulted in an increase in the LAGB fraction from 0.6% to 2.7%. WE43 joints exhibited the fewest LAGBs and the lowest dislocation density, which agrees with the statistics in KAM.

### 3.3. Mechanical Properties

**Figure 9:**
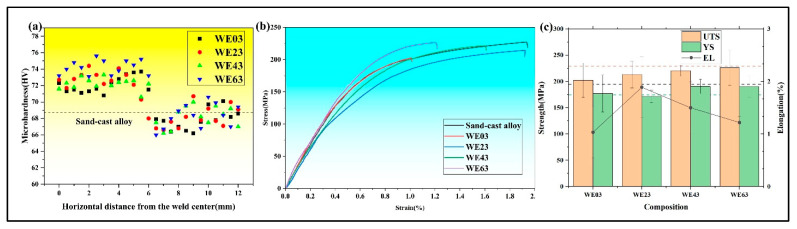
Performance of welded joints: (**a**) Vickers hardness distributions from FZ to BM, (**b**) engineering stress–strain curves, and (**c**) tensile properties of the BM and welded joints.

The Vickers hardness profiles of welded joints at different positions are shown in Figure 9a. The dashed line indicates the hardness of sand-cast BM (68.6 HV) for reference. Despite the different wire content, the width of the FZ remains constant. The hardness of the FZ is significantly higher compared to the BM. The hardness values in the FZ increased from 72.1 HV for WE03 to 72.7 HV for WE23. The hardness of the WE43 joint reached 72.2 HV. With a further increase in the Y content, the hardness of the WE63 joint is the highest at 74.2 HV, which is an increase of 8.2% compared to BM. In contrast, the hardness of the HAZ is slightly lower than that of the base metal, and its hardness is not significantly related to the filler wire content. The tensile tests were conducted to investigate the influence of filler wire content on the mechanical properties of welded joints. As illustrated in Figure 9b, there is a discernible discrepancy in the mechanical properties of different joints. Figure 9c specifically illustrates the variations in 0.2% offset yield strength (YS), ultimate tensile strength (UTS), and elongation (EL) between the welded joints as well as the BM (horizontal dotted lines). Fractures were consistently observed in the HAZ for WE23 and WE43 joints, whereas failure was confined to the FZ in the WE63 specimen. As the Y content in filler wires increased, the UTS of welded joints rose from 203 MPa to 226 MPa, achieving a joint efficiency of 99%. The 0.2% offset YS decreased from 177 MPa (WE03) to 171 MPa (WE23), then rebounded to 190 MPa (WE63). EL initially increased 90% from WE03 to a peak of WE23 (1.9%), followed by a sharp decline to 1.2% (WE63).

### 3.4. Fracture Behavior

**Figure 10:**
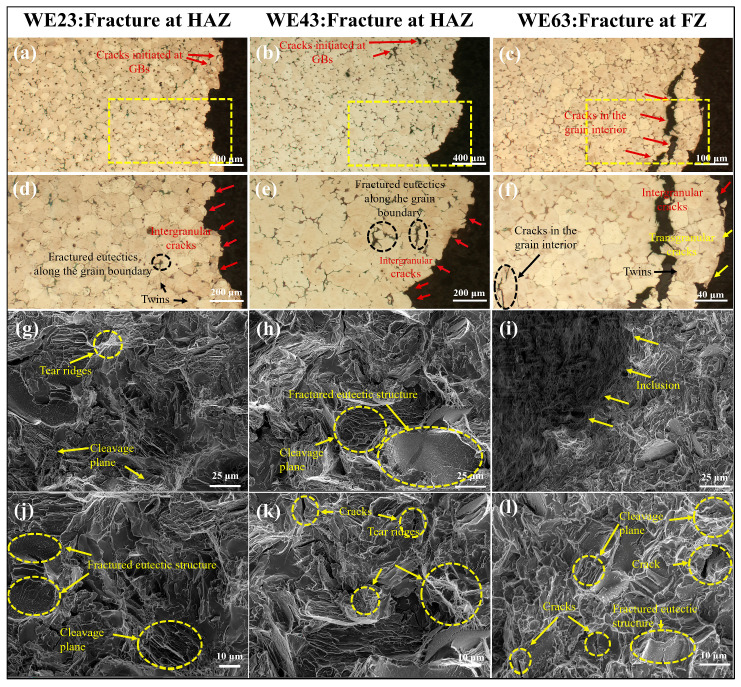
OM of the longitudinal section (**a**–**c**) and SEM of fracture surface (**g**–**i**): (**d**–**f**); and (**j**–**l**) are magnified views of selected regions in (**a**), (**b**), (**c**) and (**g**), (**h**), (i), respectively.

The transverse optical micrographs of the tensile fracture surfaces are shown in Figure 10a–f. The welded joints of WE23 and WE43 are fractured in the HAZ, while the welded joint of WE63 is fractured in the FZ. The crack initiated from the eutectic structure along the grain boundary, as shown in the black dotted circle. During loading, the continuous network of eutectics induced the occurrence of crack initiation, which harms tensile properties [22,29]. The fracture surfaces of the welded joints of WE23 and WE43 showed a predominantly intergranular fracture pattern, with evident cracks along the secondary phase boundaries. In contrast, the welded joint of WE63 exhibits coexisting transgranular and intergranular fracture features, with extensive secondary cracking.

The SEM images of fractured surfaces from different welded joints are presented in Figure 10g–l. Owing to distinct fracture locations across joints produced with varied filler wires, the fracture morphologies exhibit heterogeneous characteristics. The fracture surfaces of WE23 and WE43 joints were dominated by intergranular fracture features. These surfaces consist primarily of tear ridges and cleavage planes, along with fragmented secondary phases-collectively indicating brittle fracture behavior. This morphology corresponds to the limited elongation observed in both alloys. Notably, the weld surface of WE43 contained sparse microcracks (arrowed in Figure 10k), corroborating their marginally lower ductility relative to WE23. In contrast, the fracture surface of WE63 comprised cleavage planes, fractured secondary phases, and extensive microcracking that directly correlates with microcracks observed via optical microscopy. Crucially, extensive oxidized inclusion zones were identified at the fracture interface, directly contributing to its inferior elongation performance.

## 4. Discussion

### 4.1. Microstructural Evolution

#### 4.1.1. Evolution of the Grain

As shown in Figure 5, the FZ of the joints predominantly consisted of equiaxed grains, attributed to the combined effects of thermal undercooling and constitutional supercooling. Zhao, H. D. et al. have recalculated the Mg-Y phase diagram [30]. Obtained through both the diffusion couple technique and the alloy method, the results indicated that the maximum solubility of Y in the α-Mg matrix is 4.7 at.% (15.3% wt.%) Y. This implies that before the content of Y attains the solid solubility limit of Y in Mg, with the increase in the content of Y, the liquidus of the Mg-Y alloy gradually declines, thereby leading to different degrees of undercooling for alloys with varying Y content. Under the classical nucleation theory, whether stable nuclei can form hinges on the competition between the driving force associated with the liquid-to-solid phase transition (volume free energy) and the energy needed for the creation of a new interface. As for heterogeneous nucleation, the free energy barrier can be written as

(1) $$∆G=\left(\frac{16\pi }{3}\right)\left(\frac{\sigma^{3}}{\Delta s_{f}\Delta T^{2}}\right)\;f\left(\theta \right)$$

where σ is the interfacial energy of the new surface, Δ*s_f_* is the entropy of fusion, Δ*T* is the undercooling below the liquidus temperature, and θ is the wetting angle between the liquid phase and the heterogeneous nucleation substrate [31]. Based on Equation (1), increasing the degree of thermal undercooling can lower the free energy barrier for nucleation. Consequently, for Mg-Y-RE alloys with Y content below 15.3 wt.% (the solid solubility limit in α-Mg), an increase in the Y content leads to a gradual decrease in the liquid phase temperature, which is directly related to the degree of undercooling. The liquid-phase temperatures of the WE03, WE23, WE43, and WE63 were simulated using JMatPro software (CompuTherm LLC, 8401 Greenway Blvd Suite 248, Middleton, WI, USA), and the results are summarized in Table 3. The liquidus temperature progressively decreased from 647 °C (WE03) to 630 °C (WE63) with increasing Y content-a trend that closely matched the liquidus projection extrapolated from relevant phase diagrams. Consequently, the degree of thermal undercooling within the FZ varied significantly among filler wires. With increasing Y content in the FZ, assuming no other constraints, the resultant grain size sequence should theoretically follow: d_WE03_ < d_WE23_ < d_WE43_ < d_WE63_.

Although increasing the Y content would lead to a less significant degree of thermal undercooling, constitutional supercooling induced by RE elements constitutes another critical factor governing grain refinement. The solute contribution to grain size reduction can be quantified via the growth restriction factor (GRF):

(2) $$Q=\sum m_{i}C_{i}\left(k_{i}-1\right)$$

where *i* denotes solute elements, *m_i_* is the liquidus slope, *C_i_* is the solute concentration, and *k_i_* is the equilibrium partition coefficient. For Y solute, *m* (*k* − 1) = 1.7 [32]. Consequently, increasing Y content in the Mg matrix enhances Y-driven constitutional supercooling, thereby promoting grain refinement. If only the effect of constitutional supercooling is considered, the resultant grain size sequence should theoretically follow: d_WE03_ > d_WE23_ > d_WE43_ > d_WE63_. Owing to the combined effects of thermal undercooling and constitutional supercooling, grain coarsening happened in the WE43-welded joint.

#### 4.1.2. Evolution of the Second Phase

**Figure 11:**
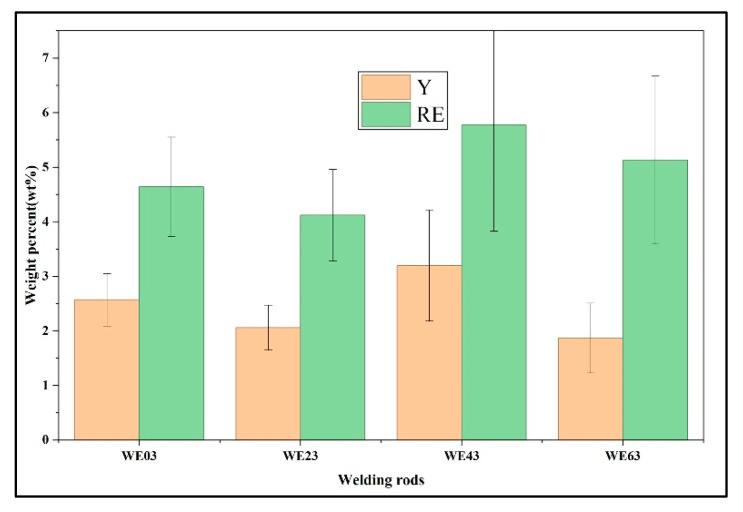
Correlation between Y content in filler wires and solute concentration within the α-Mg matrix of the FZ.

The evolution of second phase is related to the grain size and solute content. The average concentrations of Y, as well as the total RE content (consisting of Gd, Nd, and Y), were calculated to assess the relationship between the Y content in the filler wire and the actual solute content in the FZ matrix. The results are presented in Figure 11, demonstrating a nonlinear correlation. Notably, welds welded with WE43 filler wire exhibited peak concentrations of both Y (3.2 wt.%) and total RE solutes (5.8 wt.%) within the magnesium matrix. During the transition from WE03 to WE23 filler wires, the solute content in the matrix decreased significantly. As established before, this reduction is primarily attributed to the diminished degree of thermal undercooling with increasing Y content, which prolongs solute diffusion time and promotes preferential segregation of RE elements into secondary phases. Consequently, despite an overall increase in nominal solute content, the dominant factor governing solute distribution shifted to undercooling-controlled kinetics. When transitioning from WE23 to WE63 filler wires, the total matrix solute content increased, though Y solubility slightly decreased. This suggests a metastable equilibrium between undercooling effects and solute accumulation. Critically, the rise in RE solute concentration correlates with the destabilization of Mg_5_ (Gd, Y) intermetallic compounds. The similar atomic radii of Gd (1.80 Å) and Y (1.78 Å) facilitate direct lattice substitution, allowing elevated Y content to substitute for Gd atoms from the Mg_5_ (Gd, Y) lattice [33]. This atomic-scale replacement results in the release of Gd solutes, thereby increasing unbound RE concentration.

For welds produced with WE43 filler wire, the peak concentrations of both Y and rare RE solutes in the magnesium matrix are primarily attributed to anomalous grain coarsening. Grain size regulates nucleation via solute diffusion. Coarsened grains expand the width (w*) of the solute suppression nucleation (SSN) zone; the relationship is quantified as follows:

(3) $$w*=\frac{∆T_{fg}+∆T_{c}-∆T}{∆T_{c}}\delta_{C}$$

where w* is indicated as the effective width of the SSN zone, Δ*T_c_* is constitutional supercooling, Δ*T* is the thermal undercooling, Δ*T_fg_* is the critical undercooling for free growth, *δ_c_* is the width of solute diffusion zone. Furthermore, the width of solute diffusion zone *δ_c_* can be determined by:

(4) $$\delta_{C}=\frac{2D}{V_{t}}$$

where the dendritic tip growth velocity, denoted as *V_t_*, is inversely proportional to the grain radius *R_g_*, and *D* is the solute diffusion coefficient in the liquid [34,35]. Therefore, an increase in grain radius induces an increase in dendritic tip growth velocity, which in turn leads to a widening of the solute diffusion zone. Consequently, this enlargement of the solute diffusion zone causes an expansion of the SSN zone. Thus, an increase in the width of the SSN affects the formation of regions with localized solute enrichment. The solute concentration of the liquid *C_L_* can be expressed by the following equation:

(5) $$C_{L}={C_{L}}^{*}-\frac{W}{\delta_{C}}\left({C_{L}}^{*}-C_{0}\right),0\leq w\leq \delta_{C}$$

where the variable *w* signifies the distance originating from the dendrite tip, C_L_* corresponds to the solute concentration in the liquid phase at the dendrite tip itself, and *C*_0_ signifies the nominal composition of the alloy. As the width of the SSN increases, the transverse solute concentration gradient decreases; this in turn expands the extent of solute-enriched region [36]. That is the reason why the welds produced with WE43 filler wire manifest the largest solute concentration.

**Figure 12:**
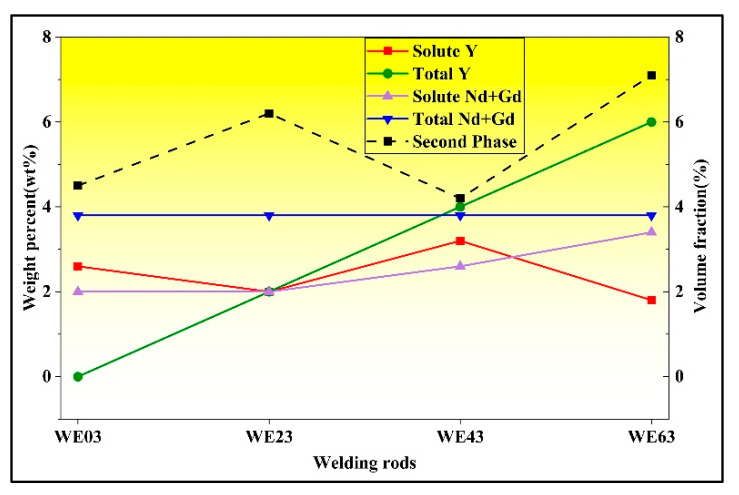
Weight percent of solute and volume fraction of second phases in FZ with different welding wires.

Furthermore, the solute concentration within the FZ influences the volume fraction of secondary phase particles. The volume fraction of secondary phases exhibits a non-monotonic trend with increasing Y content. As shown in Figure 6, when WE43 is used as the filler wire, the FZ exhibits the lowest secondary phase volume fraction of 4.2%, below the 4.5% observed in the WE03 joint. The volume fraction reaches its peak at 7.1% with the application of the WE63 filler wire. Figure 12 illustrates the weight percent of solute and volume fraction of second phases in different FZ. The observed eutectic evolution corresponds closely to the deviation between solute Y content and total Y content. Interestingly, the WE03 joint showed a deviation from the expected trend. This deviation is likely attributable to the fact that the actual composition of the FZ results from a mixture of the welding wire and the BM, whereas the current model does not account for the contribution from the BM. In addition, it can be observed that as the Y content increases, the concentrations of Gd and Nd solutes continue to rise, owing to the ‘substitution effect’, which is consistent with the previous EDS statistics.

### 4.2. Strengthening Mechanism

As illustrated in Figure 9, an increase in Y content leads to an improvement in YS. Moreover, different welding wires result in varying fracture locations. Therefore, understanding the strengthening mechanisms in welded joints is critical for optimizing joint performance. The YS of magnesium alloys primarily consists of five major strengthening mechanisms: Mg-matrix strengthening (*σ_Mg_*), grain boundary strengthening (*σ_gb_*), dislocation strengthening (*σ_d_*), solid solution strengthening (*σ_ss_*), and the second phase strengthening (*σ_sp_*) [19]. The joint welded with WE23 wire serves as an exemplary case for analyzing individual strengthening contributions, as it maintains considerable YS while exhibiting the highest elongation among the specimens.

The YS and hardness of BM are 175 MPa and 69 HV, respectively. The BM was produced by gravity sand casting with extremely slow cooling rates, resulting in negligible solid solubility of rare earth elements in the matrix, as well as ignorable dislocation density [37]. Therefore, the contribution of the strengthening mechanism for BM can be expressed as:

(6) $$\sigma_{y}^{BM}=\sigma_{Mg}+\sigma_{gb}^{BM}+\sigma_{sp}^{BM}$$

where *σ_Mg_* is the contribution of Mg matrix, which is equal to 11 MPa [38]. The effect of grain refinement on strength increment *σ_gb_* can be expressed regarding the Hall-Petch relation [39,40]:

(7) $$\sigma_{gb}=k_{y}d^{-\frac{1}{2}}$$

where *k_y_* is the Hall-Petch constant of pure Mg matrix, reported to be 164 MPa μm^1/2^, d refers to the average grain size (47.5 μm) [38,41]. Based on our calculations, $\sigma_{gb}^{BM}$ is 23.8 MPa, thus providing $\sigma_{sp}^{BM}$ with 140.2 MPa.

As for FZ of the WE23-welded joint, the YS and hardness are 171.6 MPa and 72 HV, respectively. According to the statistical analysis in Figure 8, the role of solid solution strengthening and dislocation strengthening cannot be neglected. The YS of FZ can be written as:

(8) $$\sigma_{y}^{FZ}=\sigma_{Mg}+\sigma_{gb}^{FZ}+\sigma_{sp}^{FZ}+\sigma_{d}^{FZ}+\sigma_{ss}^{FZ}$$

Following the calculation process in BM, the contribution of Mg matrix and grain refinement (d = 15.6 μm) is calculated to be 11 MPa and 41.5 MPa. For *σ_d_*, it can be represented as [19]:

(9) $$\sigma_{d}=M\alpha GB\rho^{\frac{1}{2}}$$

where *M* is the Taylor factor (4.2), α is a constant of 0.3, ρ is the dislocation density, *G* is the shear modulus of Mg (17.7 GPa), and *B* is the Burgers vector for Mg (0.3197 nm). The dislocation density can be calculated using the formula [39,42]:

(10) $$\rho =\frac{\pi \theta }{90\mu B}$$

where *θ* is the average KAM value (0.45°), *μ* the step size of EBSD mapping (1.3 μm). Consequently, the contribution of dislocation strengthening (*σ_d_*) is equal to 43.8 MPa.

In Mg-Gd-Y system, the solid solution strengthening (*σ_ss_*) can be determined by [41]:

(11) $$\sigma_{ss}={\left(\sum_{i}{k_{i}}^{\frac{1}{n}}c_{i}\right)}^{n}$$

where n is a constant (taken as 0.5), *c_i_* is the solute concentration, and *k_i_* is the strengthening constant for solute *i* [41]. Based on EDS statistical analysis, only the solid solution effects of Y, Nd, and Gd in the alloy were considered. According to previous reported data, *k_Gd_* = 683 MPa (at%)^−1/2^, *k_Y_* = 737 MPa (at%)^−1/2^ and *k_Nd_* = 432 MPa (at%)^−1/2^ [43]. EDS data showed that the proportion is 2 wt.% (equal to ~0.55 at.%), 0.6 wt.% (equal to ~0.10 at.%), 0.2 wt.% (equal to ~0.03 at.%) for Y, Nd, and Gd, respectively. The contribution of the solid solution can be predicted as 57.7 MPa. However, since the fracture of the WE23 joint occurs in the HAZ, the strengthening mechanisms in the FZ cannot be predicted using the same approach as for the second phase strengthening in the BM. Previous studies have revealed that different strengthening mechanisms contribute comparably to both hardness and YS [19,44]. Therefore, the YS of the FZ was estimated using hardness measurements as a reference.

For the BM, the contributions of strengthening mechanisms to property enhancement are 6.3% for Mg-matrix strengthening (*σ_Mg_*), 13.6% for grain boundary strengthening (*σ_gb_*), and 80.1% for second phase strengthening (*σ_sp_*). The contributions of different strengthening mechanisms to the hardness of BM can be quantitatively assessed as follows:

(12) $${HV}_{Mg}=69HV\times 6.3\%=4.3HV$$

(13) $${HV}_{gb}^{BM}=69HV\times 13.6\%=9.4HV$$

(14) $${HV}_{sp}^{BM}=69HV\times 80.1\%=55.3HV$$

As for FZ of WE23 joints, the contribution of Mg-matrix strengthening to hardness should also be 4.3 HV, which accounts for 6.0% of the total hardness contribution. Therefore, based on the proportional contribution of Mg-matrix strengthening mechanism to hardness, the nominal YS of the FZ can be derived through the following analysis:

(15) $$\sigma_{y}^{FZ}=11MPa÷\frac{4.3HV}{72HV}=184.2MPa$$

Then the contribution of the second phase strengthening can be calculated to be 30.2 MPa.

In HAZ, the thermal cycle induced by heat input led to the dissolution of second phases [40]. Therefore, similar to FZ, the YS of HAZ can be expressed as:

(16) $$\sigma_{y}^{HAZ}=\sigma_{Mg}+\sigma_{gb}^{HAZ}+\sigma_{sp}^{HAZ}+\sigma_{d}^{HAZ}+\sigma_{ss}^{HAZ}$$

The calculation method for the HAZ is consistent with the previous approach. The contribution of the Mg matrix is calculated to be 11 MPa, contributing to 6.4% of the total YS. For grain boundary strengthening, the average grain size is 60.3 μm, so the *σ_gb_* is 21.1 MPa with a ratio of 12.3%. The dislocation strengthening in HAZ is estimated as 19.1 MPa (average KAM value: 0.46°, step size: 7.0 μm), accounting for 11.1% of the *σ_y_*. According to EDS analysis, the solute concentrations in the HAZ matrix are quantified as 4.3 wt.% (equivalent to ~1.2 at.%) Y, 2.5 wt.% (equivalent to ~0.4 at.%) Nd and 1.3 wt.% (equivalent to ~0.2 at.%) Gd. Therefore, the contribution of solid solution strengthening for HAZ can be predicted as 90.5 MPa, with a proportion of 52.7%.

**Figure 13:**
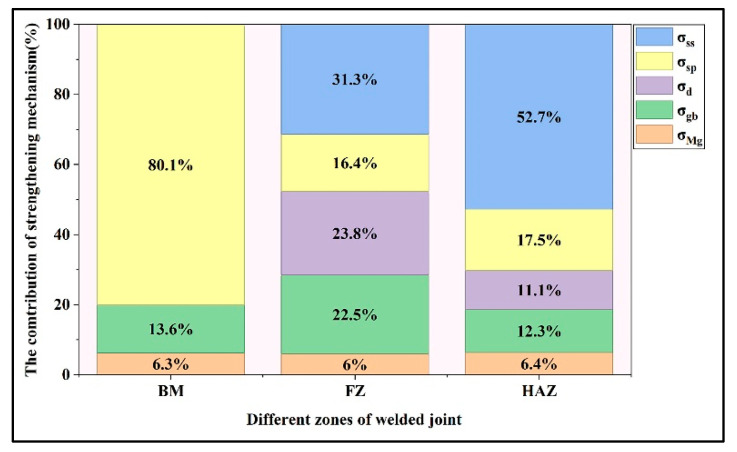
Strengthening contributions in different regions of the WE23 joint.

A systematic comparison of the contribution ratios of different strengthening mechanisms to YS across the weld joint is presented in Figure 13, with that of BM shown as a reference. The distribution of strengthening mechanisms is consistent with previous research [19]. For BM, the main strengthening mechanism is the second phase strengthening, resulting from a large proportion of eutectics. As for FZ, dramatic grain refinement leads to a significantly enhanced contribution from grain boundary strengthening. While for HAZ, owing to the grain coarsening, the contribution of grain boundaries weakened. The dissolution of secondary phases induced by thermal cycle reduces second phase strengthening but results in more contribution of solid solution strengthening.

## 5. Conclusions

In this work, the WE43 alloy was welded with welding wires with different Y content. The effect of welding wire on the weld joint quality was evaluated in terms of microstructure and mechanical properties. The main conclusions are as follows:

(1) With increasing Y content in the welding wire, the second phase in FZ transforms from a semi-continuous to a more continuous network, accompanied by more severe inclusions. The grain size of FZ initially increased, reaching a maximum grain size of 24 µm for the WE43 joint, and then decreased with further Y content.
(2) Increasing Y content in welding wire leads to a weaker effect of thermal undercooling. However, constitutional supercooling induced by more Y elements in FZ promotes grain refinement. Abnormal grain coarsening occurs with WE43 filler wire due to the combined effects of thermal undercooling and constitutional supercooling.
(3) The fracture occurs at the HAZ for joints of WE03, WE23, and WE43. All of them show brittle fracture features, while joints using WE43 show more microcracks. However, the joints of WE63 fracture at FZ, and exhibit extensive microcracking and oxidized inclusion zones.
(4) Although the UTS reached the maximum when using welding wire of WE63, the joints welded with WE23 exhibited a minimal reduction in overall performance. Relative to the sand-cast alloy, the property losses were 7.0%, 2.0%, and 2.6% for UTS, YS, and EL, respectively.
(5) The joint of WE23 wire serves as an exemplary case for analyzing strengthening contributions. Second phase strengthening serves as the primary strengthening mechanism in BM. With grain refinement in FZ, a greater proportion of grain boundary strengthening contributes to the strength. However, the dominant strengthening mechanism for HAZ was second phase strengthening, due to its large number of eutectics and precipitates.

## Figures and Tables

**Table 1:** Actual chemical composition of the materials (wt.%).

Material	Element					
	Y	Nd	Gd	Zn	Zr	Mg
Base material	3.99	2.46	1.29	0.20	0.48	Bal.
WE03	0.00	2.53	1.22	0.19	0.46	Bal.
WE23	2.05	2.51	1.29	0.17	0.47	Bal.
WE43	3.97	2.50	1.26	0.18	0.52	Bal.
WE63	6.04	2.54	1.27	0.20	0.53	Bal.

**Table 2:** The process parameters of welding.

Welding Current (A)	Tungsten Electrode Diameter (mm)	Filler Wire Diameter (mm)	Welding Speed (mm/s)	Ar Flow Rate (L/min)	Wire Feeding Speed (mm/s)
160	2.4	5	2	10	4

**Table 3:** The liquidus temperature simulated by the JMatPro software.

Welding Wires	Liquidus Temperature
WE03	647 °C
WE23	642 °C
WE43	640 °C
WE63	630 °C

## Data Availability

The original contributions presented in this study are included in the article. Further inquiries can be directed to the corresponding author.
